# Laryngo-tracheobronchial amyloidosis^☆^^☆☆^

**DOI:** 10.1016/j.bjorl.2020.07.006

**Published:** 2020-09-09

**Authors:** Juan Pablo Uyaguari, Pedro Jose Quizhpe, Evelise Lima

**Affiliations:** a Universidade de São Paulo, Hospital de Clínicas, Instituto do Coração, Serviço de Endoscopia Respiratória, São Paulo, SP, Brazil; b Universidade de São Paulo, Hospital de Clínicas, Instituto do Coração, Serviço de Pneumologia, São Paulo, SP, Brazil

Dear Editor,

We read the article by Caporrino Neto J et al., which calls attention to the clinical diversity of laryngeal amyloidosis, a localized variant of amyloidosis, representing up to 1% of benign laryngeal tumors.^1^ We would like to highlight the importance of assessing the entire airway in cases of aerodigestive amyloidosis.

We reported a case of a 52-year-old man with dysphonia for 7 months. He denied dyspnea and had no comorbidities. The physical examination, pulmonary function test and computed tomography (CT) of the neck and chest showed no alterations.

During direct laryngoscopy, yellowish infiltrative lesions were observed in the nasopharynx and epiglottis (Fig. 1 A and B), suggestive of amyloidosis. Due to the observed alterations, we chose to perform a tracheobronchial tree evaluation, and similar lesions were identified in the entire length of the trachea (Fig. 1C) and in the bronchial subsegments (Fig. 1D). Biopsies of the epiglottis and right main bronchus lesions confirmed the diagnosis of laryngo-tracheobronchial amyloidosis (LTBA).

**Fig. 1:**
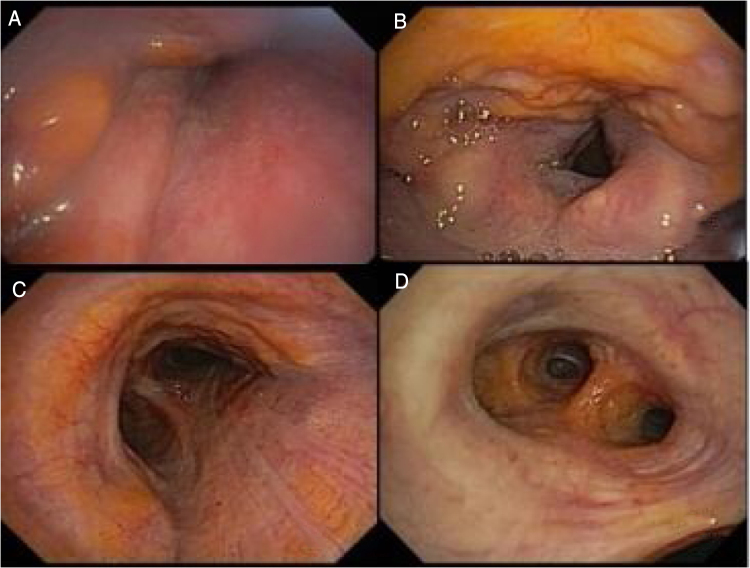
Tracheal lesions.

Primary LTBA is a rare disease, characterized by submucosal plaques or nodules of amyloid deposits in the airways, which can be localized or multifocal. It represents only 0.5 % of all symptomatic tracheobronchial lesions and is rarely associated with systemic amyloidosis.^2^, ^3^, ^4^

Symptoms are nonspecific, such as coughing, dyspnea, wheezing, hemoptysis and dysphonia, which can delay diagnosis or be mistaken by other respiratory illnesses. Pulmonary function testing and neck and chest computed tomography results may be normal.^4^, ^5^

Endoscopic visualization is the main diagnostic tool and the biopsy is the gold standard for the diagnosis.^5^ The presence of homogeneous extracellular amyloid deposits, eosinophilic amorphous material and green birefringence under polarization microscopy with Congo Red staining confirm the diagnosis.^5^

Although it is not closely associated with systemic amyloidosis, the latter should be ruled out through serum and urine electrophoresis tests to detect monoclonal proteins, together with an echocardiography to investigate cardiomyopathy secondary to amyloid deposits.^4^, ^5^

There is no specific treatment. Mechanical excision, balloon dilation, stenting, argon plasma coagulation, laser, cryotherap, electrocautery and tracheostomy are alternatives that have been described for the treatment of symptomatic patients.^3^, ^4^, ^5^

In conclusion, LTBA is a rare disease that should be considered in patients with subacute symptoms of airway obstruction without an adequate response to treatment and should be investigated in patients with aerodigestive amyloidosis. The diagnostic method of choice is respiratory endoscopy with the identification of yellowish infiltrative lesions, since the definitive diagnosis is attained through the biopsy of lesions.

## Conflicts of interest

The authors declare no conflicts of interest.
